# Characterization of the *Glehnia littoralis* Non-specific Phospholipase C Gene *GlNPC3* and Its Involvement in the Salt Stress Response

**DOI:** 10.3389/fpls.2021.769599

**Published:** 2021-12-09

**Authors:** Li Li, Naiwei Li, Xiwu Qi, Yang Bai, Qiutong Chen, Hailing Fang, Xu Yu, Dongmei Liu, Chengyuan Liang, Yifeng Zhou

**Affiliations:** ^1^Institute of Botany, Jiangsu Province and Chinese Academy of Sciences (Nanjing Botanical Garden Mem. Sun Yat-Sen), Nanjing, China; ^2^College of Forestry, Nanjing Forestry University, Nanjing, China

**Keywords:** *Glehnia littoralis*, halophyte, non-specific phospholipase C, salt stress, lipid

## Abstract

*Glehnia littoralis* is a medicinal halophyte that inhabits sandy beaches and has high ecological and commercial value. However, the molecular mechanism of salt adaptation in *G. littoralis* remains largely unknown. Here, we cloned and identified a non-specific phospholipase C gene (*GlNPC3*) from *G. littoralis*, which conferred lipid-mediated signaling during the salt stress response. The expression of *GlNPC3* was induced continuously by salt treatment. Overexpression of *GlNPC3* in *Arabidopsis thaliana* increased salt tolerance compared to wild-type (WT) plants. *GlNPC3*-overexpressing plants had longer roots and higher fresh and dry masses under the salt treatment. The *GlNPC3* expression pattern revealed that the gene was expressed in most *G. littoralis* tissues, particularly in roots. The subcellular localization of GlNPC3 was mainly at the plasma membrane, and partially at the tonoplast. GlNPC3 hydrolyzed common membrane phospholipids, such as phosphotidylserine (PS), phosphoethanolamine (PE), and phosphocholine (PC). *In vitro* enzymatic assay showed salt-induced total non-specific phospholipase C (NPC) activation in *A. thaliana GlNPC3*-overexpressing plants. Plant lipid profiling showed a significant change in the membrane-lipid composition of *A. thaliana GlNPC3*-overexpressing plants compared to WT after the salt treatment. Furthermore, downregulation of *GlNPC3* expression by virus-induced gene silencing in *G. littoralis* reduced the expression levels of some stress-related genes, such as *SnRK2*, *P5SC5*, *TPC1*, and *SOS1*. Together, these results indicated that *GlNPC3* and GlNPC3-mediated membrane lipid change played a positive role in the response of *G. littoralis* to a saline environment.

## Introduction

*Glehnia littoralis* Fr. Schmidt ex Miq. is a medicinal and edible plant in the Umbelliferae family. *Glehnia littoralis* is rich in coumarins, coumarin glycosides, phospholipids, and polysaccharides (Yuan et al., 2002). The peeled and dried roots and rhizomes of *G. littoralis* are commonly used as a traditional Chinese herbal medicine for moistening the lungs, removing phlegm, relieving cough, curing gastrointestinal disorders, recovering from surgery, and immunoregulation, as well as for their anti-inflammatory properties (Huang et al., 2012; Li et al., 2020). Moreover, the tender leaves of *G. littoralis* are edible as a vegetable. Therefore, the demand for *G. littoralis* as a clinical medication and health care product is always high. *Glehnia littoralis* is a perennial halophyte that grows on sandy beaches in Northern Pacific countries and regions, such as eastern China, Japan, the Korean Peninsula, Russia, and the United States (Li et al., 2018). A previous study elucidated how *G. littoralis* adapts to high-salinity environments by analyzing its anatomical and morphological characteristics, such as the secretory trichomes and thick cuticle cover on leaves (Voronková et al., 2011), but the molecular mechanism of salt adaptation in *G. littoralis* remains largely unknown.

The phospholipid bilayer of the plasma membrane is the first barrier to the external environment. Phospholipases, including phospholipase A (PLA_1_, sPLA_2_, and pPLA), phospholipase C (PLC), and phospholipase D (PLD) hydrolyze membrane phospholipids, and affect the structure and stability of the cellular membrane and signaling responses to environmental stimuli (Wang, 2005; Scherer et al., 2010; Hong et al., 2016). The products of phospholipases, such as phosphatidic acid (PA) and inositol 1,4,5-trisphosphate, are cellular messengers that regulate various biological processes (Testerink and Munnik, 2005; Wang et al., 2006; Tang et al., 2007). PLD and PLC/diacylglycerol kinases (DGK) play a major role in the stress-induced generation of PA (Munnik and Testerink, 2009). The activities of the phospholipases are induced rapidly, coupled with a highly dynamic level of PA, in response to hormones, as well as abiotic and biotic stimuli (Bargmann and Munnik, 2006; Li et al., 2006; Peters et al., 2010). PA acts as a cellular mediator by binding with some regulatory proteins, anchoring these proteins to the membrane, modulating their catalytic activities, and altering membrane structure (Wang et al., 2006; Testerink and Munnik, 2011). PA-interacting proteins include various kinases, transcription factors, phosphatases, and other target proteins during cellular processes (Zhang et al., 2004, 2012; Yu et al., 2010; Kim et al., 2019; Shen et al., 2019, 2020).

Non-specific phospholipase C (NPC) is a subtype of PLC that hydrolyzes common membrane phospholipids, such as phosphatidylcholine (PC), phosphatidylethanolamine (PE), and phosphatidylserine (PS), to produce *sn*-1,2-diacylglycerol (DAG) and a corresponding phosphorylated headgroup. DAG is phosphorylated by DGK to generate PA. Unlike PI-PLC (another PLC subtype), which is found widely in animals, plants, and bacteria, NPC is found only in bacteria and plants, and has distinct evolutionary features (Nakamura, 2014; Nakamura and Ngo, 2020). In the model plant *Arabidopsis thaliana*, six NPCs have been identified, most of which have been reported to be involved in multiple biological processes. NPC4 is associated with the plasma membrane, and the recombinant protein of NPC4 shows enzymatic activity toward PC and PE (Nakamura et al., 2005). NPC4 is involved in the plant response to abscisic acid (ABA), auxin, phosphate deficiency, hyperosmotic conditions, and salt and Al stresses (Nakamura et al., 2005; Gaude et al., 2008; Peters et al., 2010; Wimalasekera et al., 2010; Kocourková et al., 2011; Yang et al., 2021). NPC4 and NPC3 are important in brassinolide-mediated signaling in root development (Wimalasekera et al., 2010). NPC4 is also involved in phosphosphingolipid hydrolysis and remodeling in the presence of a phosphate deficiency during root growth (Yang et al., 2021). NPC1 plays a vital role in plant heat tolerance (Krčková et al., 2015). NPC1 is localized at secretory pathway compartments, such as the endoplasmic reticulum or Golgi apparatus, so is NPC2. NPC2 expression is suppressed after *Pseudomonas syringae* attack, and NPC2 is involved in plant immune responses, such as PTI, ETI, and SA (Krčková et al., 2018). The double-mutant *npc2npc6* displays a lethal homozygous phenotype and a defect in the heterozygous gametophyte (Ngo et al., 2018). NPC5 is a cytosolic protein. NPC5 and its derived DAG mediate lateral root development under salt stress, and NPC5 is involved in galactolipid accumulation during phosphate limitation (Gaude et al., 2008; Peters et al., 2014). The localization of NPC6 is present in both chloroplast and microsomal fractions, but not in cytosolic or nuclear fractions (Cai et al., 2020). NPC6 promotes seed oil content and enhances PC and galactolipid turnover to TAG (Cai et al., 2020).

The important roles of NPCs in other plant species have been gradually revealed following the NPC findings in *A. thaliana*. For example, Cai et al. (2020) reported that BnNPCs affect seed mass and yield, and that BnNPC6.C01 is positively associated with seed oil content in oilseed rape. In rice, five OsNPCs are identified. The cytosolic and membrane-associated OsNPC1 modulates silicon distribution and secondary cell wall deposition in nodes and grains, affecting mechanical strength and seed shattering (Cao et al., 2016). OsNPC6 is involved in mesocotyl elongation in rice. *OsNPC6*-overexpressing plants exhibit a shorter mesocotyl than the wild-type (WT) and *npc6* mutant (Yang et al., 2021). Yang et al. (2021) also indicated that all five OsNPCs showed plasma membrane localization. These studies suggest that plant NPCs are involved in numerous biological processes, although the functional properties of most plant NPCs await exploration. In the current study, we cloned and identified *GlNPC3* from *G. littoralis*, which played a positive role in the salt stress response.

## Materials and Methods

### Plant Materials and Growth Conditions

The *G. littoralis* plants used in this study were obtained and grown as described by Li et al. (2018). The seedlings were subjected to salt (200mM NaCl), drought (20% PEG 6000), or hormone [100μM ABA or 100μM methyl jasmonate (MeJA)] stress treatments for 0, 6, or 24h. The shoots and roots were sampled separately at different time points in each treatment.

*Arabidopsis thaliana* Columbia (Col-0, WT) and its transgenic seeds were sown on Murashige and Skoog (MS) medium with vitamins (1% sucrose and 1% agar) and supplemented with 75mM NaCl for the salt stress treatment. The plants were grown in a vertical position (for root growth) in a growth chamber under a 14-h-light (23°C)/10-h-dark (20°C) photoperiod. The roots were measured using the ImageJ software (NIH, Bethesda, MD, United States).

### *In silico* Characterization of GlNPC3

The coding sequence (CDS) of *GlNPC3* was amplified from *G. littoralis* cDNA using the *GlNPC3-F1* and *GlNPC3-R1* primers. The locations of the exons and introns were defined using GSDS V2.0.^1^ The protein domains were identified with Pfam database.^2^ The *GlNPC3* upstream promoter sequence was amplified using the Genome Walking Kit (Takara, Dalian, China) with the *SP1*, *SP2*, and *SP3* primers. The plant regulatory elements in the *GlNPC3* promoter region were obtained from the PlantCARE database.^3^ All the primers are listed in Supplementary Table S1. The gene, protein, CDS, and a 2,070-bp promoter sequence of *GlNPC3* are shown in Data Sheet 1.

### Vector Construction and Plant Transformation

To generate the *GlNPC3*-overexpressing construct (pSuper::GlNPC3), the CDS was amplified using the *GlNPC3-F2*/*GlNPC3-R2* primers. The product was cloned into the pSuper1300 binary vector, which was pCAMBIA1300 containing a *Super* promoter (Ni et al., 1995; Cao et al., 2013), and digested with *Xba*I/*Sal*I. To generate the Promoter_GlNPC3_::GUS construct, a 2,070-bp promoter sequence was amplified using the *Pro-GlNPC3-F/Pro-GlNPC3-R* primers and cloned into the PMV plant binary vector (Hua et al., 2021). The primers are listed in Supplementary Table S1.

*Arabidopsis thaliana* transformation was performed according to the floral dip method (Clough and Bent, 1998). The harvested seeds were selected on MS medium containing 25mg/L hygromycin until the T3 generation. The homozygous lines were used for the experiments.

### Subcellular Localization of GlNPC3

The CDS sequence of *GlNPC3* was inserted into the pSuper1300GFP vector using the *GlNPC3-F3* and *GlNPC3-R3* primers to generate pSuper::GFP-GlNPC3 recombinant plasmid. The N-terminus of GlNPC3 was translationally fused to GFP under the control of *Super* promoter. A plasma membrane (PM) marker (AtCBL1n-OFP) and tonoplast marker (AtCBL6-OFP) were used as described by Batistič et al. (2010). The plasmids were introduced into *Agrobacterium tumefaciens* (GV3101) for transient expression in tobacco (*Nicotiana benthamiana*), and the tobacco leaves were infiltrated as described previously (Li et al., 2017). Fluorescence was observed using an LSM 780 confocal microscope (Zeiss, Jena, Germany).

### GUS Staining

Histochemical staining for GUS expression in transgenic *A. thaliana* was performed according to the method of Jefferson (1987).

### Protein Expression and Enzyme Activity Assays

The *GlNPC3* CDS was cloned into the pCold I vector (Takara) with a His-tag at its N-terminus and transformed into *E. coli BL21* (DE3). Isopropyl β-D-thiogalactopyranoside (0.1mM) was added and the culture was incubated at 15°C for 24h to induce expression of the His-GlNPC3 fusion protein. The protein was purified with TALON® Metal Affinity Resin (Clontech, Palo Alto, CA, United States) according to the manufacturer’s protocol.

GlNPC3 activity was assayed as described previously (Nakamura et al., 2005; Peters et al., 2010) with some modifications. The purified His-GlNPC3 protein or total plant protein was incubated for 1h at 37°C in a 500μl reaction mixture (50mM Tris-HCl, pH 7.3, 50mM NaCl, and 5% glycerol) in the presence of a sonicated micellar suspension of 200μM of the fluorescent head-group-labeled lipid substrate. The lipid substrates 18:1 NBD-PS (810198C), 18:1 NBD-PE (810145P), and 18:1 Cy5-PC (850483C) were obtained from Avanti Polar Lipids, Inc. (Alabaster, AL, United States). The reaction was stopped by vigorous vortex with 1ml of ethyl acetate and 0.75ml of 0.45% NaCl. Centrifuge the reaction at 1,500*g* for 10min and remove supernatant (water-soluble phase) to a new tube carefully, do not suck middle layer (protein) and lower organic phase (lipid). The water-soluble phase was used to determine fluorescence using a microplate reader (SpectraMax ID5; Molecular Devices, San José, CA, United States) with the following parameters: NBD, excitation at 460nm and emission at 534nm; Cy5, excitation at 648nm and emission at 662nm.

### RNA Extraction and Quantitative Real-Time PCR

RNA extraction and quantitative real-time PCR (RT-qPCR) were performed according to protocols described previously (Li et al., 2020). The primers for RT-qPCR are listed in Supplementary Table S1.

### Lipid Analysis

The lipid extraction and electrospray ionization tandem mass spectrometry (ESI-MS/MS) analyses were performed as described previously (Welti et al., 2002; Devaiah et al., 2006).

### Virus-Induced Gene Silencing in *G. littoralis*

Virus-induced gene silencing (VIGS) was performed as described previously (Liu et al., 2002). A 409-bp fragment of *GlPDS* (phytoene desaturase gene) was amplified from the *G. littoralis* CDS using the *PDS-F/PDS-R* primers and constructed using pTRV II as the positive control. Similarly, 199- and 200-bp fragments of the CDS within *GlNPC3* were cloned into pTRVII using the *vigs-F1*/*vigs-R1* and *vigs-F2*/*vigs-R2* primers. The fragments were designed using the SGN VIGS tool^4^ (Fernandez-Pozo et al., 2015). The recombinant vectors were transferred into *A. tumefaciens* strain GV3101. Suspensions of *A. tumefaciens* containing recombinant vectors were mixed with suspension of *A. tumefaciens* containing pTRV I (helper vector) for infiltration. The silencing effect was monitored in the filtered leaves after 14days by RT-qPCR. The silencing phenotype (photobleaching phenotype) of VIGS-PDS was used as an indicator of VIGS efficiency. All primers are listed in Supplementary Table S1. The fragments used for the VIGS assay are listed in Data Sheet 2.

## Results

### Cloning and Identification of *GlNPC3*

**Figure 1:**
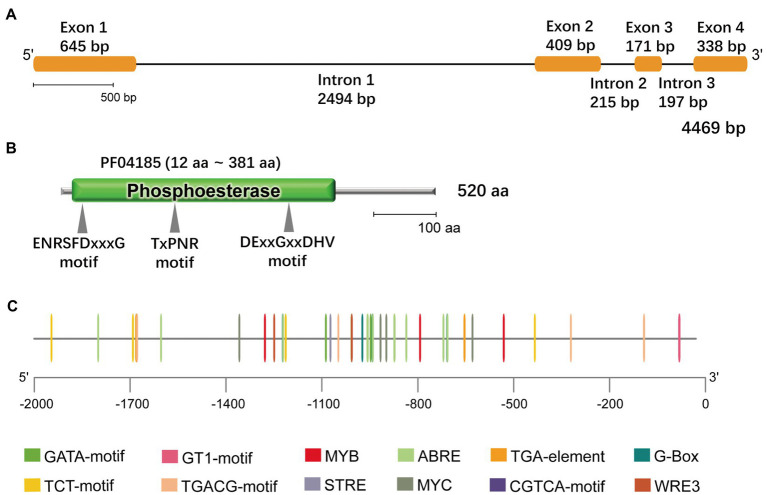
Bioinformatics features of *GlNPC3*. **(A)** *GlNPC3* exons and introns obtained through GSDS V2.0. The yellow rectangles represent exons, and the black lines represent introns. **(B)** Schematic representation of the GlNPC3 protein; the phosphoesterase domain (Pfam entry PF04185) and conserved domains are indicated. **(C)** Plant regulatory elements in the *GlNPC3* promoter region were obtained from the PlantCARE database.

The phospholipase-mediated lipid signaling pathway plays an important role in the abiotic stress response in plants. Based on the *G. littoralis* salt-related transcriptome data obtained in our previous study (Li et al., 2018), we cloned a *NPC* that showed a positive response to salt treatment in *G. littoralis*. This gene contained an open reading frame of 1,563bp encoding a protein of 520 amino acids (Figure 1A). Phylogenetic analysis of this NPC revealed a close relationship to *Daucus carota* L. DcNPC3; therefore, we named this gene *GlNPC3* (Supplementary Figure S1). GlNPC3 contained a phosphoesterase domain with three motifs commonly present in plant NPCs (Figures 1B,C). To better understand the function of GlNPC3, the 2,070-bp *GlNPC3* promoter sequence was cloned. We analyzed the main regulatory *cis*-elements in the *GlNPC3* promoter region using the PlantCARE database (Figure 1C; Data Sheet 1). Some regulatory elements are involved in plant hormone and abiotic stress responses, such as the light-responsive G-box, GT1-motif, TCT-motif, and GATA-motif; hormones responsive to AREB, the CGTCA-motif, and the TGA-motif; and the stress-responsive MYB, MYC, and STRE.

### *GlNPC3* Plays a Positive Role in the Salt Stress Response

**Figure 2:**
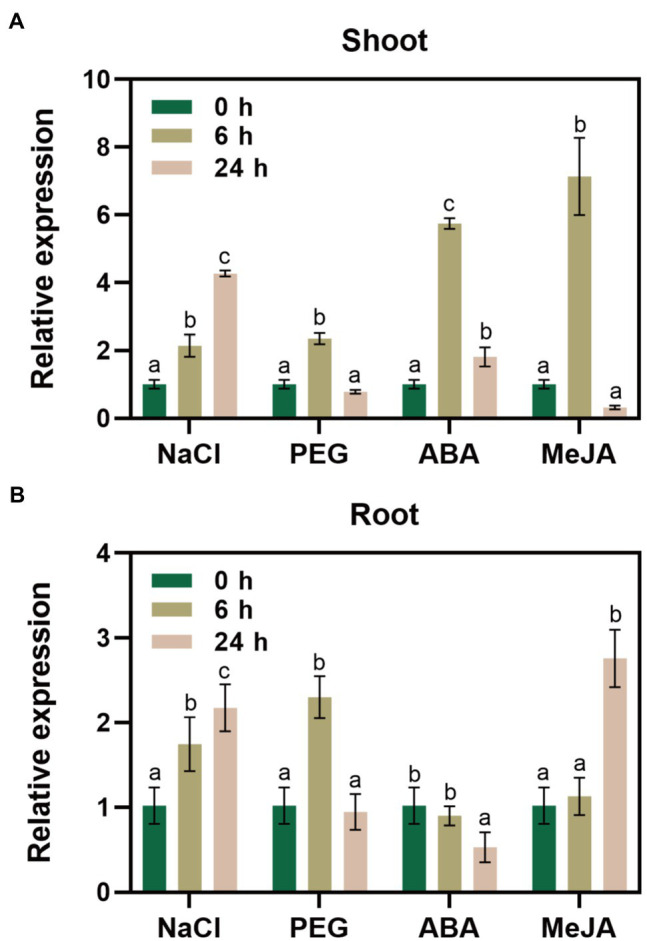
Relative expression levels of *GlNPC3* under different stress conditions. *GlNPC3* expression levels in shoots **(A)** and roots **(B)** under different stress conditions. *Glehnia littoralis* seedlings were subjected to salt (200mM NaCl), drought (20% PEG 6000), or hormone (100μM ABA or 100μM MeJA) treatments for 0, 6, or 24h. The shoots and roots of *G. littoralis* were harvested at the indicated time intervals for RNA extraction. *GlCYP2* was used as the internal control. Data represent means three biological replicates with three pooled plants each ± SD (three technical replicates per biological replicate). Different letters indicate statistically significant difference in each treatment (*p*<0.05, Duncan’s multiple range test).

**Figure 3:**
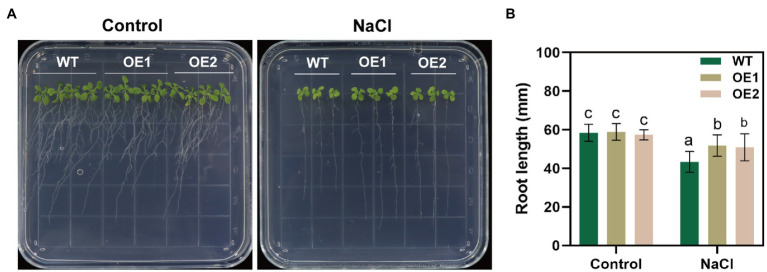
Effect of NaCl on root length in the *GlNPC3*-overexpressing plants. **(A)** Phenotypes of the wild-type (WT) and transgenic *Arabidopsis thaliana* during salt stress. The seeds were grown on agar plates supplemented with 0mM (control) or 75mM NaCl for 14days. Representative photographs of the plants are shown. **(B)** Root length of 14-day-old seedlings. Data represent mean±SD, *n*=48. Different letters above each bar indicate a significant difference (*p*<0.05, Duncan’s multiple range test).

We performed RT-qPCR to reveal changes in *GlNPC3* gene expression when the *G. littoralis* seedlings were exposed to ABA, MeJA, NaCl, or osmotic stress (PEG). The results showed that *GlNPC3* positively responded to these treatments in shoots and roots, and the expression of *GlNPC3* increased continuously within 24h after the salt treatment (Figure 2). The trends in *GlNPC3* expression determined by RT-qPCR were consistent with those shown in our previous RNA-seq data (Li et al., 2018). As a stable *G. littoralis* genetic transformation system has not been established, we transformed recombinant plasmid (pSuper::GlNPC3) into *A. thaliana* (Col-0) to verify the biological function of *GlNPC3* in the salt stress response. Homozygous transgenic lines were isolated and exogenous *GlNPC3* expression was evaluated in the transgenic lines by reverse transcription PCR (Supplementary Figure S2). It was found that exogenous *GlNPC3* was over-expressed in transgenic lines OE1 and OE2 (Supplementary Figure S2). The OE lines showed no obvious phenotype when grown under normal conditions compared to WT plants. However, when plants were grown in medium supplemented with 75mM NaCl, the main root length of the OE lines were significantly longer than that of the WT (Figure 3). Moreover, soil-cultivated plants were exposed to the salt treatment. Four-week-old plants were watered with Hoagland’s culture solution containing 100mM NaCl, and the concentration was maintained for 10days. The fresh and dry masses were higher in OE lines than the WT under the salt treatment (Supplementary Figure S3). The leaf size of the WT decreased compared to that of the OE lines. Thus, these results suggest a positive role for *GlNPC3* in *G. littoralis* salt tolerance.

### GlNPC3 Expression Pattern and Localization

**Figure 4:**
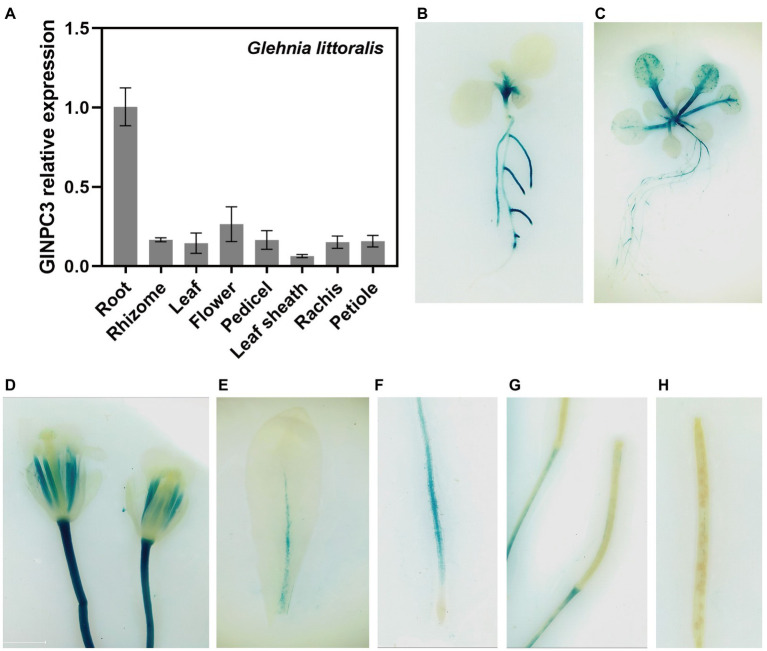
Expression pattern of *GlNPC3* in plants. **(A)** Tissue-specific expression analysis of *GlNPC3* in *G. littoralis*. Data represent means±SD of three biological replicates with three pooled tissues (plants) each. *GlEXP1* was used as the internal control. **(B–H)** Histochemical GUS-staining of transgenic *A. thaliana* (Promoter_GlNPC3_::GUS). Histochemical GUS-stained 4-day-old seedling **(B)**, 10-day-old seedling **(C)**, flower **(D)**, old leaf **(E)**, root **(F)**, and siliques **(G,H)** are shown.

**Figure 5:**
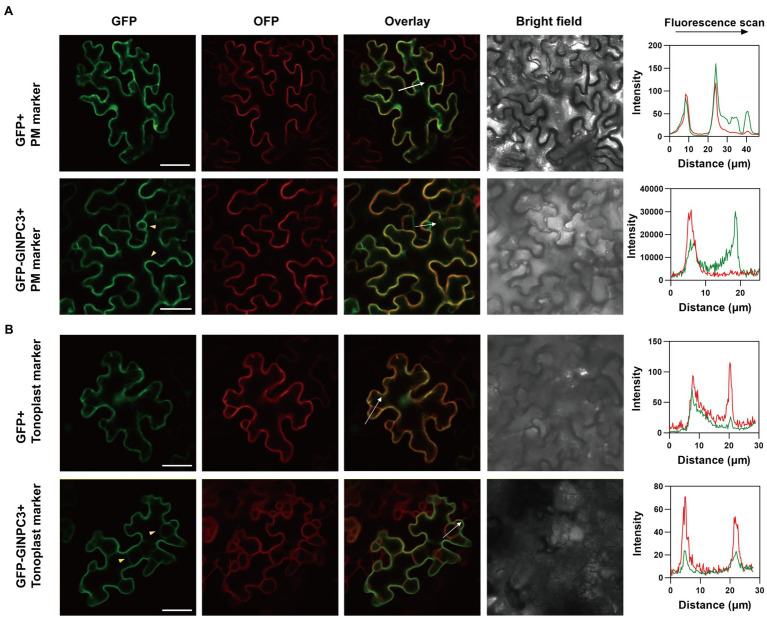
Subcellular localization of GlNPC3 in leaf epidermal cells of *N. benthamiana*. **(A)** Upper row, GFP empty vector was co-expressed with a plasma membrane (PM) marker. Lower row, GFP-GlNPC3 fusion protein was co-expressed with the PM marker. PM marker, AtCBL1n-OFP. Bars, 40μm. The fluorescence intensity profiles along the direction of white arrows were obtained using Zen (blue edition). The yellow arrow heads indicate the PM and tonoplast localization of GlNPC3. **(B)** Upper row, GFP empty vector was co-expressed with the tonoplast marker. Lower row, GFP-GlNPC3 fusion protein was co-expressed with the tonoplast marker. Tonoplast marker, AtCBL6-OFP. Bars, 40μm. The fluorescence intensity profiles along the direction of white arrows were obtained using Zen (blue edition). The yellow arrow heads indicate the tonoplast localization of GlNPC3.

We examined the tissue expression pattern and subcellular localization to further understand the function of GlNPC3 in *G. littoralis*. RT-qPCR revealed that *GlNPC3* was expressed in most tissues, including roots, rhizomes, leaves, flowers, pedicels, leaf sheath, the rachis, and the petioles (Figure 4A). *GlNPC3* was highly expressed in roots, at a level more than three times higher than in other tissues (Figure 4A). A GUS construct driven by the *GlNPC3* native promoter (Promoter_GlNPC3_::GUS) was transformed into *A. thaliana*. As shown in Figures 4B–H, GUS staining occurred in the seminal and lateral roots, hypocotyl, leaves, stamens, and stems. Promoter_GlNPC3_::GUS displayed a high level of expression in leaves at the *A. thaliana* eight-leaf stage; however, little expression was detected at the four-leaf stage and in old leaves (Figures 4B,C,E). The expression of *GlNPC3* varied spatiotemporally. Moreover, the expression of Promoter_GlNPC3_::GUS also responded to exogenous NaCl, mannitol or the hormones treatments in the roots of transgenic *A. thaliana* seedlings (Supplementary Figure S4). To explore the subcellular localization of GlNPC3, GFP-GlNPC3 fusion protein was co-expressed with a PM marker or tonoplast marker tagged with orange fluorescent protein (OFP) in *N. benthamiana* leaves (Batistič et al., 2010). The distribution of green and orange fluorescence indicated that GlNPC3 was predominantly localized at the plasma membrane, with some staining of the tonoplast (Figure 5).

### Biochemical Characterization of GlNPC3

**Figure 6:**
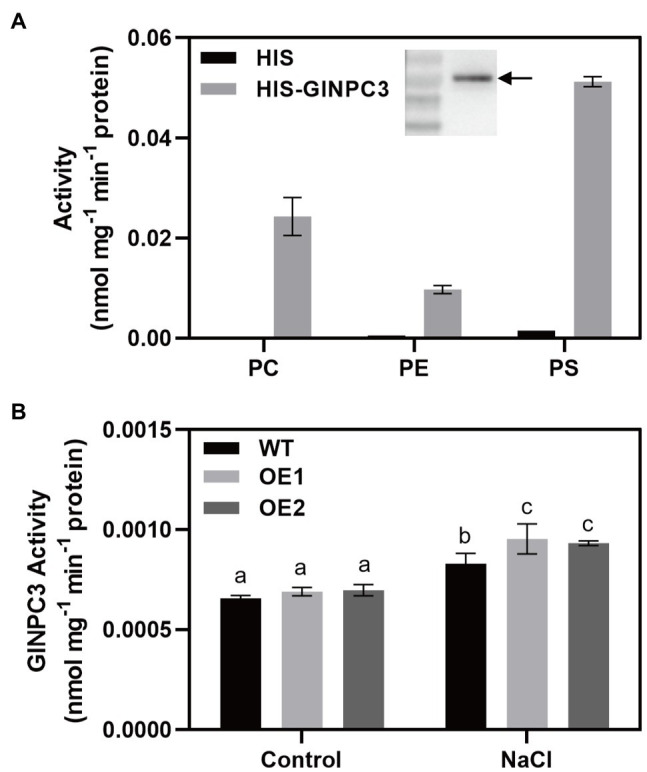
GlNPC3 activity assay *in vitro*. **(A)** His-GlNPC3 activity assay in the presence of different fluorescent head-group-labeled lipid substrates. Data are mean±SD of three measurements. Inset, immunoblotting assay of His-GlNPC3 fusion protein with the His antibody. Arrow indicates the protein. **(B)** Total non-specific phospholipase C (NPC) activities of WT and transgenic *A. thaliana*. Total protein was extracted from *A. thaliana* seedlings exposed or not to 75mM NaCl for 24days. Fluorescent head group-labeled phosphotidylserine (PS; 200μM) was used as the substrate. Data represent means±SD three biological replicates. Different letters above each bar indicate significant differences (*p*<0.05, Duncan’s multiple range test).

To determine the biochemical characteristics of GlNPC3, the GlNPC3 protein fused with a His-tag at its N-terminus (His-GlNPC3) was expressed in *E. coli* and subjected to an *in vitro* enzymatic assay (Figure 6A, inset). GlNPC3 activity was assayed using various fluorescent head group-labeled phospholipid substrates, including NBD-PS, NBD-PE, and Cy5-PC. Phospholipases hydrolyze lipid substrates and produce a soluble group with fluorescence, which is easy to detect with a microplate reader. In our results, His-GlNPC3 fusion protein displayed twice the hydrolyzing activity toward the PS substrate compared to PC, and five times the activity compared to PE (Figure 6A). As a negative control, His-tag protein (empty vector) displayed little enzymatic activity toward these substrates (Figure 6A). Next, we extracted total protein from WT and *GlNPC3*-overexpressing lines (OE1 and OE2) for enzymatic activity assay, respectively. Total NPC activity was assayed using the PS substrate. The WT and OE lines showed similar NPC activity under the control condition. NPC activity was significantly induced by salt treatment (Figure 6B). Total NPC activity was higher in the OE lines after the salt treatment compared to the WT. These results suggest that GlNPC3 contributes to the overall NPC activity in plants during the salt stress response.

### Overexpression of *GlNPC3* Alters Membrane-Lipid Composition in Transgenic Plants

**Figure 7:**
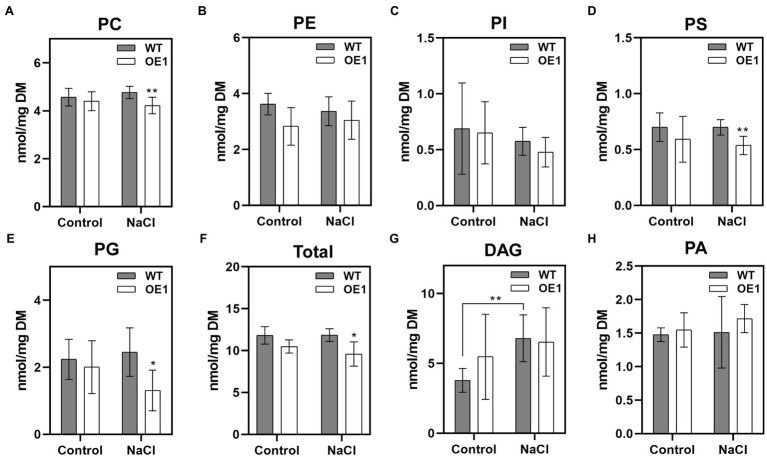
Effect of *GlNPC3* overexpression on membrane lipid contents in *A. thaliana*. **(A–H)** Comparison of lipid changes between the WT and OE1. Total lipids were extracted from 10-day-old medium-grown *A. thaliana* seedlings that were exposed or not to 150mM NaCl for 45min. Lipids were quantified by electrospray ionization tandem mass spectrometry (ESI-MS/MS). Total lipids refer to the amounts of PC, PE, PI, PS, and PG. Data represent means±SD of five replicates (each replicate contained 15~20 plants). Asterisks indicate significant differences from the WT: ^*^p≤0.05; ^**^p≤0.01, Duncan’s multiple range test. DM, dry mass.

Membrane lipids affect the stability of membranes, and are involved in various cellular responses to abiotic and biotic stresses. To investigate the mechanism underlying GlNPC3 lipid metabolism in response to salt stress, we determined the lipid composition of transgenic *A. thaliana* using ESI-MS/MS-based lipid profiling (Figure 7). Under normal condition, there was no significant difference in the levels of PC, PE, phosphatidylinositol (PI), PS, phosphatidylglycerol (PG), DAG, and PA between WT and *GlNPC3*-overexpressing seedlings (OE1; Figures 7A–H). After 45min of salt treatment, the lipid composition changed to varying degrees (Figure 7). The levels of PC, PS, and PG in OE1 decreased obviously compared to that of WT. DAG content increased significantly in WT after exposure to the salt treatment, however, no significant difference occurred in OE1 (Figure 7G). Generally, NPC hydrolyzed glycerolipids to generate DAG, and DAG simultaneously generated PA through DGK kinases. Then, we analyzed PA content. In our result, there was no significant difference in the level of total PA between salt-treated and non-treated plants. Furthermore, a molecular species analysis revealed the lipid composition of the plants (Supplementary Figure S5). The molecular species in the seedlings with a higher mass spectral signal were 34:3, 34:2, 36:4, 36:5, and 36:6 for PC, PE, PI, DAG, and PA; 42:2, 42:3, 40:2, and 40:3 for PS; and 34:4, 34:3, and 32:1 for PG. Among them, both 36:4 PA and 34:6 PA in OE1 were significantly higher than that in WT, and the same DAG species were increased in OE1 under normal condition (Supplementary Figure S5). The changes in major lipid molecular species (Supplementary Figure S5) were almost consistent with those shown in sum (Figure 7).

### Downregulation of *GlNPC3* Affects the Transcript Levels of Some Stress-Related Genes

**Figure 8:**
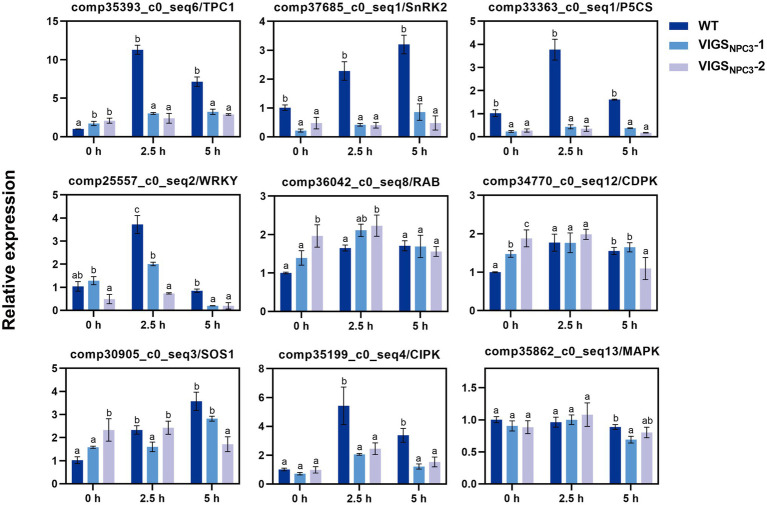
Relative expression of stress-related genes in *G. littoralis* WT, VIGS_NPC3_-1, and VIGS_NPC3_-2 leaves. For NaCl treatment, the pot-cultured *G. littoralis* plants were watered with Hoagland’s culture solution containing 200mM NaCl. Data represent means±SD three biological replicates. Different letters above each bar indicate significant differences (*p*<0.05, Duncan’s multiple range test). *GlEXP1* was used as the internal control.

It was difficult to obtain transgenic *G. littoralis* plants, so we used VIGS to transiently silence the *GlNPC3* gene in *G. littoralis* leaves for rapid functional analysis. First, the endogenous *G. littoralis* phytoene desaturase gene (*GlPDS*), which causes photobleaching, was used as a positive control to assess VIGS efficiency (Supplementary Figure S6). The photobleaching phenotype was different from senescence (Supplementary Figure S6A). Photobleaching was confined to infiltrated leaves, perhaps because of the unique morphology of *G. littoralis*, which has extremely short rhizomes and a long petiole, and a basal petiole that expands into the sheath. Thus, only infiltrated leaves were used for the RNA extraction and RT-qPCR analysis. Then, we selected two interfering fragments (*VIGS_NPC3_-1* and *VIGS_NPC3_-2*) of the *GlNPC3* CDS for VIGS-silencing. The specificity of gene silencing was detected in infiltrated leaves (Supplementary Figures S6B–D). *GlNPC3* expression was almost 50 and 70% reduced in the injected leaves of VIGS_NPC3_-1 and VIGS_NPC3_-2, respectively (Supplementary Figure S6B). We also checked two of the *GlNPC3* homologous genes. Their expression levels were not reduced as much as that of *GlNPC3* (Supplementary Figures S6C,D). RT-qPCR analysis was performed to investigate whether the GlNPC3-mediated lipid changes were accompanied by a gene expression response. According to the RT-qPCR results, most of the stress-related genes were significantly induced by the NaCl treatment (Figure 8). In particular, transcription of *GlSnRK2* (comp37685_c0_seq1), *GlP5CS* (comp33363_c0_seq1), *GlWRKY* (comp25557_c0_seq2), *GlSOS1* (comp30905_c0_seq3), and *GlCIPK* (comp35199_c0_seq4) increased more than 3-fold in response to the salt stress, while the transcription of *GlTPC1* (comp35393_c0_seq6) increased more than 10-fold. The induction of most of the tested genes was significantly inhibited in the VIGS_NPC3_-1 and VIGS_NPC3_-2 leaves (Figure 8).

## Discussion

*Glehnia littoralis* is a medicinal halophyte that grows in coastal habitats. There is an inevitable association between saline adaptability and the accumulation of active medicinal ingredients in *G. littoralis*. Shu et al. (2019) indicated that the accumulation of furocoumarins was increased by NaCl stress in *G. littoralis*. Few studies have been reported on the salt tolerance mechanism of *G. littoralis*. In our previous study, we performed a comprehensive transcriptome analysis of the response of *G. littoralis* to salt stress, and obtained a large number of differentially expressed genes involved in basic metabolism, secondary metabolism, transportation, and signal transduction (Li et al., 2018). We cloned and identified a differentially expressed NPC gene named *GlNPC3* (Figure 1). RT-qPCR showed that the expression level of *GlNPC3* increased continuously upon salt treatment (Figure 2). The characterization of *A. thaliana GlNPC3*-overexpressing plants showed that overexpression of *GlNPC3* enhanced the salt tolerance of *A. thaliana*. Therefore, we performed a series of detailed experiments to elucidate the involvement of *GlNPC3* in the salt stress response in *G. littoralis*.

Similar to most *NPCs*, *GlNPC3* contained a conserved phosphoesterase domain necessary for esterase activity and NPC catalytic activity (Figure 1 and Supplementary Figure S1). In *A. thaliana*, AtNPC1, AtNPC2, and AtNPC6 contain a signal peptide at the C-terminus, but AtNPC3, AtNPC4, and AtNPC5 lack it (Pokotylo et al., 2013). GlNPC3 contains no signal peptide and was clustered with AtNPC3, AtNPC4, and AtNPC5 in the phylogenetic analysis. The 50–100 amino acids at the C-terminus of NPCs are the most variable regions among NPC subfamilies. These regions may be responsible for various molecular functions and subcellular localization of different NPCs (Pokotylo et al., 2013). The gene expression changes under the different treatments showed that *GlNPC3* responds to various stresses (Figure 2). Our analysis of the *GlNPC3* promoter sequence also showed the variety of stress-related regulatory elements in the *GlNPC3* promoter sequence, suggesting that *GlNPC3* might be involved in a variety of transcriptionally regulatory processes. Moreover, we found that the expression of the GUS reporter driven by the *GlNPC3* promoter in the roots of *A. thaliana* was upregulated after NaCl, mannitol or the hormone treatments during 2h (Supplementary Figure S4). However, the expression of *GlNPC3* did not increase at 6 and 24h after ABA treatment in the root of *G. littoralis* (Figure 1B). We supposed that the response of *GlNPC3* to ABA might be earlier or later than the time points we analyzed in *G. littoralis*.

In previous studies, *AtNPCs* were expressed at different levels in the roots (Peters et al., 2010), and some AtNPCs were involved in the regulation of root development. For example, AtNPC2 and AtNPC6 are required for root growth in *Arabidopsis* (Ngo et al., 2019). AtNPC4 is involved in the root response to salt stress (Kocourková et al., 2011), and AtNPC5 mediates lateral root development during salt stress (Peters et al., 2014). Our results showed that *GlNPC3* was expressed at the highest levels in the roots of *G. littoralis*, followed by flowers (Figure 4). Expression of the GUS reporter driven by the native *GlNPC3* promoter in *A. thaliana* exhibited a similar pattern with that of *G. littoralis*. However, there were some differences compared to *G. littoralis*. For example, Promoter_GlNPC3_::GUS was highly expressed in the stem of *A. thaliana*, while *G. littoralis* had extremely short rhizomes and the expression level in rhizomes was similar to that in the petiole. Moreover, the roots of *A. thaliana GlNPC3*-overexpressing plants were longer than that of the WT under the salt treatment (Figure 3). Based on the growth characteristics of *G. littoralis*, the roots of *G. littoralis*, which are deeply embedded in the beach, are not only the main medicinal parts but also play a positive role in salt tolerance. Therefore, GlNPC3 may be related to the response of roots to salt stress.

The subcellular localization of GlNPC3 was mainly at the plasma membrane and partially at the tonoplast, different from other NPCs (Figure 5). Both AtNPC1 and AtNPC2 are localized at the endoplasmic reticulum and Golgi apparatus (Krčková et al., 2015; Ngo et al., 2018), and AtNPC2 is localized at the plastid (Ngo et al., 2018). AtNPC4 is associated with the plasma membrane, whereas AtNPC5 localizes to the cytosol (Gaude et al., 2008; Peters et al., 2014). The subcellular localization of the NPC isoforms is generally related to enzyme activity and biological function (Nakamura and Ngo, 2020). The localization of GlNPC3 suggests that it may hydrolyze phospholipids at the plasma membrane and tonoplast, and may be involved in the lipid-mediated signaling pathway or in the lipid metabolism.

Previous studies have reported that NPCs hydrolyze a variety of common lipids, such as PC, PE, and PS (Pokotylo et al., 2013). Changes in NPC substrates and products affect intracellular lipid signaling messengers, and the composition and remodeling of membrane phospholipids, and are involved in a series of cellular responses (Nakamura et al., 2005; Gaude et al., 2008; Krčková et al., 2015; Ngo et al., 2018). We analyzed GlNPC3 catalytic activity using fluorescent head group-labeled lipids as substrates. Compared to previously reported NPCs, the hydrolytic activity of GlNPC3 in the presence of PS was higher than that in the presence of PC *in vitro* (Figure 6A); however, the PS content in the plants was significantly lower than that of PC and PE (Figures 7A,B,D). Because of the limitation associated with the use of a single molecular species substrate (18:1) in the *in vitro* assay, detailed analysis of phospholipids was needed in the plants. In this study, NPC enzyme activity increased in *GlNPC3*-overexpressing plants after the *in vitro* salt treatment (Figure 6B). Then, the changes in phospholipid content of the WT and OE1 were determined under salt stress (Figure 7). The common phospholipids, such as PC, PS, and PG, decreased significantly in OE1 compared to the WT under NaCl treatment, suggesting that these lipids may be hydrolyzed by GlNPC3 in plant. Direct measurement of the DAG product showed that the DAG contents increased significantly in the WT at 45min after the salt treatment. However, no significant difference was observed between WT and OE1. Previous studies showed that DAG was phosphorylated to PA under hyperosmotic stress conditions (Arisz et al., 2009; Munnik and Testerink, 2009; Nakamura, 2014). We then analyzed PA content; however, the increase in PA did not occur at 45min after the salt treatment. Why OE plants had less substrates during NaCl treatment, and no significant increase in DAG and PA levels? On one hand, the lipid molecules involved in hydrolysis and synthesis are not limited to these classes that we analyzed. For example, PC also contributes to DAG-mediated triacylglycerol (TAG) synthesis (Bates et al., 2009; Lu et al., 2009). Moreover, Cai et al. (2020) found that AtNPC6 promoted polar glycerolipid and galactolipid [monogalactosyldiacylglycerol (MGDG) and digalactosyldiacylglycerol (DGDG)] conversion to TAG production. In contrast, MGDG and DGDG were converted to DAG by monogalactosyldiacylglycerol synthase (MGD) and digalactosyldiacylglycerol synthase (DGD). TAG and galactolipid were not measured in this study. So, we speculated that OE plants may promote polar glycerolipid turnover to DAG to form TAG during salt treatment, and affect DAG and PA levels. Under normal condition, although no significant difference was observed in DAG content between WT and OE1, OE1 still displayed more DAG content than WT (average 5.47 vs. 3.78nmol/mg DM), and less total polar lipids than WT (average 10.49 vs. 11.82nmol/mg DM; Figures 7F,G). This data suggested that *GlNPC3* overexpression contributed the generation of DAG under the normal condition. Together, lipid profiles suggested over-accumulation of GlNPC3 affected the proportion of different membrane lipids and the spatial and electronic properties of membrane, conferring salt tolerance to plants.

On the other hand, the “time limitation” might affect the determination of PA and DAG content. A significantly salt-induced increase in PA and DAG between WT and OE line may occur in the other time points during salt treatment. PA is metabolized rapidly in plants, requiring appropriate time points and rigorous operation, and the extraction and determination of PA are complicated and costly (Welti et al., 2002; Shadyro et al., 2004; Nguyen et al., 2017). Because of the spatiotemporal complexity of monitoring lipid in living cells and tissues of plants, a real-time quantitative and visualized method is required. For example, Potocky et al. (2014) constructed a PA biosensor with a Spo20p-PA binding domain fusing YFP protein, which reflected PA dynamics in transiently transformed tobacco pollen tubes as shown by confocal laser scanning microscopy. Subsequently, Li et al. (2019) developed a PA-specific biosensor, PAleon, which monitors and visualizes the concentration and dynamics of PA in plant cells under abiotic stress based on Förster resonance energy transfer (FRET) technology. PA biosensors have revealed the spatiotemporal dynamics of PA in plants. Similarly, DAG, PS, PtdIns(4,5)P_2_ (phosphatidylinositol-4,5-biphosphates), PtdIns4P (phosphatidylinositol-4-phosphate), and PtdIns3P (phosphatidylinositol-3-phosphate) biosensors have been successfully used in plant cells (Vermeer et al., 2006, 2009; van Leeuwen et al., 2007; Thole et al., 2008; Yamaoka et al., 2011; Simon et al., 2014; Vermeer et al., 2017). These biosensors could be used for real-time monitoring of lipid dynamics and provide new insight into lipid metabolism in *G. littoralis*.

Finally, we analyzed the expression of some stress-related genes in WT and VIGS-silenced *G. littoralis* plants to investigate whether the GlNPC3-mediated lipid changes were associated with gene expression. The expression of *SOS1* (salt overly sensitive 1), *TPC1* (two-pore channel 1), *SnRK2* (Ser/Thr protein kinase), *P5CS* (∆1-pyrroline-5-carboxylate synthetase), *CIPK* (CBL-interacting protein kinase), and the *WRKY* transcription factor genes in *G. littoralis* was significantly upregulated under the salt treatment, but little changed after VIGS-induced *GlNPC3* silencing of the plants (Figure 8). These genes play pivotal roles in responses to multiple stresses in model plants. Among them, the vacuolar ion channel *TPC1* is responsible for a reactive oxygen species-assisted Ca^2+^ wave during the salt stress response (Evans et al., 2016). The SOS signaling pathway gene *SOS1* plays a key role in Na^+^ export (Zhu, 2001, 2016). Therefore, combined with the membrane localization of GlNPC3, these findings suggest that a GlNPC3-mediated lipid change may be accompanied by the response of stress-related genes affecting *G. littoralis* salt tolerance.

In conclusion, we cloned and identified a *NPC* (*GlNPC3*) from the medicinal halophyte *G. littoralis*, and uncovered a potentially functional connection between GlNPC3-mediated phospholipid signaling and the salt-stress response in *G. littoralis*. Further investigation is needed to determine the role of GlNPC3 in lipid remodeling, and how it is regulated by upstream mediators during the stress response. These results will provide insights into studies on plants living in special environments.

## Data Availability Statement

The original contributions presented in the study are included in the article/Supplementary Material, further inquiries can be directed to the corresponding authors.

## Author Contributions

LL and YZ designed the experiments. LL performed the experiments, analyzed the data, and wrote the manuscript. YB provided support for protein expression and enzyme activity assays. NL and QC provided support for plant growth. XQ helped in bioinformatics analysis. HF, XY, and DL provided the materials and technical support. CL and YZ revised the manuscript. All authors contributed to the article and approved the submitted version.

## Funding

This study was supported by grants from the National Natural Science Foundation of China (No. 31800272).

## Conflict of Interest

The authors declare that the research was conducted in the absence of any commercial or financial relationships that could be construed as a potential conflict of interest.

## Publisher’s Note

All claims expressed in this article are solely those of the authors and do not necessarily represent those of their affiliated organizations, or those of the publisher, the editors and the reviewers. Any product that may be evaluated in this article, or claim that may be made by its manufacturer, is not guaranteed or endorsed by the publisher.

## Footnotes

^1^ http://gsds.gao-lab.org/index.php

^2^ http://pfam.xfam.org/

^3^ http://bioinformatics.psb.ugent.be/webtools/plantcare/html/

^4^ http://vigs.solgenomics.net/
