# Supplementary material for: Characterization of the Glehnia littoralis Non-specific Phospholipase C Gene GlNPC3 and Its Involvement in the Salt Stress Response
Source: Front Plant Sci. 2021 Dec 9;12:769599. doi: 10.3389/fpls.2021.769599 (PMC8695444; doi:10.3389/fpls.2021.769599)
Supplement: Supplementary file 3 [file Data_Sheet_1.DOCX]

***GlNPC3* CDS**

ATGGCACCCGAAGCAACAAGCAACACTTATCCCATTAAAACAATAGTCCTTGTGGTCCAA

GAAAACCGCTCATTTGATCACATGCTAGGATGGATGAAATCCTTAAACAACAACATCAAT

GGTGTTACCGGAGGTGAATCCAATCCGTTATCCGCCTCCAATCCATCCGGAGGACGCGTG

TTTTTTGGTGACGGGTCTGGCTACAGCGAGCCAGACCCTGGTCACTCACTAGAAGCAACA

CATGAGCAACTTTATGGGGCTGCTTTAACTTCCACTACATTTTCGAGCAATAGTAATGAT

AATATTCGTATCCCGACAATGGAAGGATTCGCTGAACAAGCTGAGAGTATAAACAAAGGC

ATGTCTGATATTGTCATGAACGGATTCAAACCAGAAATGGTGCCAGTTTACAAAGAATTG

GTGGAAGAGTTTGCGGTTTGTGACAGGTGGTTTTCCTCGATTCCGACACTAACACAGCCT

AACAGATTGTACATACATTCGGCTACGTCTCATGGTGCAACAGAAAATGATACAAGTACG

TTGATTAAAGGGTATCCACAGAAAACCATCTTTGAGTCATTGGAAGAAGGGGGGTGCACT

TTTGGGATTTATCATCAATATCCTCCCAACACACTCTTCTTTAGGAATATGAGAAAGTTA

AAGTACTTGGACAATTACCATCAGTTTGATCTACAGTTCAAAGACCACTGTGAAAAAGGG

AAATTACCAAATTATGTGGTAATTGAAAACCGTTATTTTGACACAAAATTGTTACCCGGA

AATGATGATCACCCTGCTCATGATGTTTCGGAAGGTCAAAAATTTGTGAAACAAGTTTAT

GAAGCATTGAGATCAAGCCCCCAGTGGAATGAGATGTTGTTTCTGATTATTTATGATGAA

CATGGTGGTTTCTATGATCATGTCCCACCTCCAGTGACTGGAGTCCCCAGTCCAGATGAC

ATCGATGGTCCTGAACCATATAAATTCAAATTTGACCGACTAGGAGTTAGGGTTCCGGTA

ATCATGATTTCTCCCTGGATTGAACGAGGAACAGTATTGCATGGGCCTTCAGGACCATAT

CCAACTTCAGAGTTTGAGCATTCTTCACTTGCCGCAACTATCAAGAAGATCTTTAATCTG

AAAGACTTTTTGACAAAGAGAGATGCATGGGCTGGCACTTTTGAATGTGTTCTTAACAGG

AGTAGTCCAAGACAGGATTGTCCAGAGAAATTATCAGAGCCAGTGAAAATGCGTGATTTT

GAACCAAATGAAGATGAAGCAAAGCTAACTGATTATCAAGGAGAAATGGTACAAATGGCT

GCAACACTGAACGGTGACCATAATAAAGATATTTACCCTCACAAACTAGTTGAAAATATG

ACAGTTTCTGAAGCTGTCAAATACGTGGGAGACGCATATAAAGTTTACTGTGACGAGTGC

TCCAAAGCTAAAGAAAGTGGAGCTGATGAACATCATGTTGTTTCTTTCGAAGCTCTGGTT

CCAAAATCATCTCCAAAATCTTTTGGCCAGAAATTGTTTTCGTGCTTGATTTGTGATAAT

TAG

***GlNPC3* gene sequence**

ATGGCACCCGAAGCAACAAGCAACACTTATCCCATTAAAACAATAGTCCTTGTGGTCCAAGAAAACCGCTCATTTGATCACATGCTAGGATGGATGAAATCCCTAAACAACAACATCAATGGTGTTACCGGAGGTGAATCCAATCCGTTATCCGCCTCCAATCCATCCGGAGGACGCGTGTTTTTTGGTGACGGGTCTGGCTACAGCGAGCCAGACCCTGGTCACTCACTAGAAGCAACACATGAGCAACTTTATGGGGCTGCTTTAACTTCCACTACATTTTCGAGCAATAGTAATGATAATATTCGTATCCCGACAATGGAAGGATTCGCTGAACAAGCTGAGAGTATAAACAAAGGCATGTCTGATATTGTCATGAACGGATTCAAACCAGAAATGGTGCCAGTTTACAAAGAATTGGTGGAAGAGTTTGCGGTTTGTGACAGGTGGTTTTCCTCGATTCCGACACTAACACAGCCTAACAGATTGTACATACATTCGGCTACGTCTCATGGTGCAACAGAAAATGATACAAGTACGTTGATTAAAGGGTATCCACAGAAAACCATCTTTGAGTCATTGGAAGAAGGGGGGTGCACTTTTGGGATTTATCATCAATATCCTCCCAACACACTCTTCTTTAGGTAACATAAGTATTTTCTTGCTCACTGCATTATGCAATTTTTCTTCTTATTAAACTACGTATTTTTATGATGGATGCATAAATCTAAATACTCGCACATCAATCTCTTTGACATCTGAACCATTAACTTGTTGGGAATAGCTGGTTGTTGATATTTGAATTTTCTTTTCGGTCATCTTTCTGTTTCACTTTATTTAACATTTTTCTTTGTGTAGACGCTGGTACGCAAAAGATGTATTTTAAGGATAGTTTTATAATTTTTTTTAAGTTTTCTTTTTTTATATAAAAATTTAAACATTAAATTTTTATACAAAAAAAAAAGAAAATTTTAAAGAAAATTATGAAATTATTCTTAAAATACACATTAAAATATGTCCCTATTTCAAATGTTAAGAATCAAGTTGGGAGTATAAGACAACTAGAAATGATCTTGCGAACTAGCTCTATTAATCATGTATCATCGACTCTGTTTTTTAATCACTCTTAACAAATCTTTTGCTGAAAACATAATAACTGTTTTACATGATATTAAACAAAGTATACTATTCCTGAAAAAAAAAAAAAAAAAAAACAAAGTATACTATTGATGGAGCATCTTCCCTCTTCCGTGTAGATATTGATTTCTATAGGTGATAAAACTTGGGTGTAATTCGAGTCGAGTCGAGTTTGAGCTTTTTTTTCAAGTTTAAGCTTTTTTTTTCGAGTCGAGTCGAGCCTAAAATTTATCTAGCCTGAAATTTATGTTTGAACTCGACTGGGTTTACTATACGAGTTGAGTTCGAGCTGTTATCGAGCTTTCGAGCTTATCGAATTTTTAACCGAGCTTATCGAGTTTTTAAACGAGTTTCCGAGCTTTTATCAAGTTTTTGAGTTTTCGATTTTCGAGTTTTTCGAGTTCTTACCGAGTTTTCGAGTTTATCGGGTTCTTATCGAGTTTTCGAGTTTATCGGGCTCTTGTCGAGTTTTGACTTAAGTTCCATGAAAACTCTTTGTTTATATTATTTTCTTTTCAAGTTTAACCTAAATTTTTTTTCCGAATTTTGAAATGACAATAAACTTTAGAAATTTTACATTTTATGATTTCGATAATAAACTTTACCAATCAATAAGTAAACTTTCAGTTTATTCTATTCATATATATTTACGGATAAATTTTATCTTTATTACGAGTTATGCAATTAACTATCTAAGCATCTTAAGTAGTAAACGAATAAACGATGATACTAAGAATAATAAAATGTGAAACATTTTTTGATTGTGAGCATATATGGGTTTTATTTTTTCAAAATAGGCAAATAGGCACCCTACACCGAGAGTCATTAGTAGTTTTATAAATTTTGTTTATAATTTAATAGAAATATTGCATACAACAAGTCTATGATATTACTATCTTGCTGTTATAGTTTATAATTGTACAAAGACAAAATATATGGAAGTAAAATTCAGAAATCGAACTTGATCGCTTACAATCCAGGATCGATCAAAAGATCAAAATATTTATAAAAATGTATAATTAAATATCTCATATTCTCATATTAGAAATTTTTAATTTAAAATTTTCACTAACTATATTAATAATAGGGTTTAATGATCCTATAGTTAACTGTAGGAGAATTTCTTAAAGTTAATCAGTGAAATCAAGACACATCACATAGTTTCCTAAATAAAAAGAAGTATAAGGTAACAAATATTTAAAATTACATGATTTTAAAGGTTAACTTTAGGAAATTCTCCTAAATTTAACTGTAAGAACATTTTTCTCTTAATAATATAATATTGCACATAATATATGAAAAAAATATCAAGTTAAGTTTTAAAAATAATTTTCATAAATAGTTGAGTGATTAGACAATGAATAATAAAAACATGACTATTTTCTAAAAAAATTAAAAAAAATTAATCGAGTTCGAGTTCGAGCCGAGTCGAGTTCGAGTTTATCGAGTCGAGCTCGAGTCGAGCTCAAAAAAAACTCGGCTCGACTCGAGTTTTTTATCGAGCCCAAAAACATGTTCGAACTCGAGTTCGATAACGAGTTCGAATCGAGTCGAGCCAACTTTTTCGAGTCGAGCTGGAGTCGAGTTCGATCTGGCACGATTCAAATTACACCCCTAGATAAAACCCGCCCTAGACAAAAAAGTCGACCCGAATGAGAAAATCAAGACAATATTTTGTGTTCAAAAGCAGAACATCTGGTTTTCATAGTTCCCTTCTTTTATTGTTATTTTGAATCTGTCCCTTTATTACTATGTGTTTTCCTGTATAGGTAGTAGTTTTTTTTTTATTTAATAACTTGAGCAGATATGCTTAGATGCTCATCATTTTAAAAGGTGTAAAGAGAAAATGGAATATTGCAAGTGCGGCAGCCGACAGATTAAACGATCATTACGATATAACCATGTTCATTAATCATTATATTATGCAGTGAAATGTTGGGACAACATGCTGTGGTAAAATGTGGTTTTTATCGAATTACAAATGAAAACTTCCTTTTACAGGAATATGAGAAAGTTAAAGTACTTGGACAATTACCATCAGTTTGATCTACAGTTCAAAGACCACTGTGAAAAAGGGAAATTACCAAATTATGTGGTAATTGAAAACCGTTATTTTGACACAAAATTGTTACCCGGAAATGATGATCACCCTGCTCATGATGTTTCGGAAGGTCAAAAATTTGTGAAACAAGTTTATGAAGCATTGAGATCAAGCCCCCAGTGGAATGAGATGTTGTTTCTGATTATTTATGATGAACATGGTGGTTTCTATGATCATGTCCCTCCTCCAGTGACTGGAGTCCCCAGTCCAGATGACATCGATGGTCCTGAACCATATAAATTCAAATTTGACCGACTAGGAGTTAGGGTTCCGGTAATCATGATTTCTCCCTGGATTGAACGAGGAACAGGCGAGTCTATGATCATTATTACTTATTCATGCTTTGAAAAGAAAAACAAGGATTGAATCCATTCTTTAGTGTCCTAAAATAGATATAAAGGCTTTTATCTCGAAAAATATTACTGATATGGATGTAGTTTGCATAGGTTATATCACCTTCCAAATAATTTTAGGATGTGAACGATTTATAAACCAACTAAATCTTTCATGAGTTGGAATTTGCAGTATTGCATGGGCCTTCAGGACCATATCCAACTTCAGAGTTTGAGCATTCTTCACTTGCCGCAACTATCAAGAAGATCTTTAATCTGAAAGACTTTTTGACAAAGAGAGATGCATGGGCTGGCACTTTTGAAGGTGTTCTTAACAGGAGTAGTCCAAGACAGGATTGTCCAGGTATGCATATCTATTAAAATTTTGCATTGCATTTTAAATTTTGCTTTAAAAATATTTCCTTTTTTTTTTACCTCAAGTAAATTACCAAGACTCTTCACAATAGCCACAGATTTTAGGAGTAGGATAGACTTGGTATGGTTTAGTTTATACTAAATCAAAATTATTATTTAAGCTATGAAAATGTGAATAAAATGCAGAGAAATTATCAGAGCCAGTGAAAATGCGTGATTTTGAACCAAACGAAGATGAAGCAAAGCTAACTGATTATCAAGGAGAAATGGTACAAATGGCTGCAACACTGAACGGTGACCATAATAAAGATATTTACCCTCACAAACTAGTTGAAAATATGACAGTTTCTGAAGCTGTCAAATACGTGGGAGACGCATATAAAGTTTACTGTGACGAGTGCTCCAAAGCTAAAGAAAGTGGAGCTGATGAACATCATGTTGTTTCTTTCGAAGCTCTGGTTCCAAAATCATCTCCAAAATCCTTTGGCCAGAAATTGTTTTCGTGCTTGATTTGTGATAATTAG

**Promoter sequence (2,070-bp fragment upstream of *GlNPC3* ATG codon)**

TTCAGCGAATCCTTCCATTGTCGGGTAAAAAAAATCACATAACAACAATATCTTACTATATAAACTCATGAAAGTCTTTTTACTAACATGACATTACCCTTTTCATTTACAATTTTCCACTTCTATGTGATGTAATTTGGATATCATATCTCATCCATCTGTGGATAATTTTTGAGTACATGTATTGAAAAATATTCACGTAGAAGAACATATCAAAGTATAACTTATTACGCGCATAGAACAAGACCTAAACAAATCGATGCTCAAACTGTTTTGATTACATTTTCCAGCAGTGTTAAAATGATTCTTACCAACTTACGTCAATCAACAACTAAATATGTTGATCTTAATTAGTACTCCCTTCGTCTTATTACTCTTCCTGTTCATCACTATCACGTTTATTAATGCACACTTTTGATCGTTAATATCTTTAATTTCATATTAATATTAAATATAAAAATTTTATTATATTAAACTATTCACAAATACGAATCAAATAAAATCACTCATAATTTTATTTTATTTTATAAATTATGCATAAATTAATAATTAATTACTTATAATAAATAATATAAAAAGTCAAAACAAGAAAAATATTACGAGCCGGAGGGAGGATGAATGTTCCATCACTCTGTTTACATTTGTTCATATTGATAACTCCATATCCCGATCAGATGCCTCCTCGCCCGTTCCCACGCCTGGAAGAGGTGTATGCTGGTGGTTGGGCCTCTGCAAAACAACGCCGGGAGGTGGGTTTGAACCCGCGGCACCTCCGACGTGAGAGTAAGAGTAGGCTTTGGAGGATAAGAAAATAAGAACTAGAGTATCGAAATGTGGAGGGTGTGTGTATATGAGTGTGTAAAAGAACGTAACCCCCACACATTCCTCACCTGGTCTTATTTATAGACCAAGGATTAGGGTTCAGGGGTTGGTACCTGCAATCATGAGCGTCAGATCCCAGGTATGCGGGACACTGTATGTTGTAGATGGCCACCTGTCCAGGTGATAATTGTGGTGGCGTGTGTCGTGCTCATGTCAGCACGTGGGTTGATAGGAACGTGATTGTGCCCATCATTGGATTCTCTTATTTGTCATTATCACATGGGTCCCTCCTCATGACCTGTACGTGTCATTGTTATTAATGTGGTGTAGTGTTTTCTCACGTACCATGGGCCTGAGCTGTGGGTGCTTTGGATTGGGTCTCAACTGGGACTAGTACCAGACAAGTCTGGCATTAGCCTGGACTGAGTGCAGCCTAAGCTAGTTTATAACCTATCACGTATATGCCAACGTGATGGCTAATTTTAGGTAAATATTTTAAAGTAAAAAATTGGAAAGTGCAAACGACTAAGCTACCTAACTATTTGCATTTGTACGCAATATACATGTTGCAATATACATGTTACAAGTTGACATAAAATATTGGAATGGTCTAATGTACATTGTTGACCTCTGGCATATCATATAACCATAGATCACCGTGGGCTGCGGATGAAAAGTGACATGATCTGTCAGTCTGTCAGTGTCATGCAGTTTAAATAAATAAAGAGTTAAGTAAAGTTGTAAGAAAGGATAATATATTGAAGTAATTTTTTACAGATTAAAAGATTAAAATTATGATGAAGAGATAAGAAATTTAAGTGGAATAGAAAAAAAAAAAAATCGACCTCTGATTCGTCATCTGAATTCAATTAAATCCAATCTAATTGACCTCTAATCTAATTGATCTCTGTTTGAGTACTTGTCTGAGCTAATCATTTTTTACAGCTCGATAATTTATAGCTACAAAAGCAATAATTAGACTCTATAATTCAACAACTACATTAACTTGGCATAATTGAATATAGAATATAATCCACTTGTTTATAAAAACTCATGCAAGAGTTCTCGATATTGAAAAGTCTTGACGACTTTAATTTGATACACATTGTGCTTTCAACTTCATCAACAATTCATCTTCATCTTCCAAGCCCCGTATCAATTCAAACAAAAGAAGGAACACTAAAAGAAGAAAATTAACCCTCAAATCCAATATAACAGAAACCATCTCAAAGCCAGAAGGTAGAAAAA

**GlNPC3 protein sequence**

MAPEATSNTYPIKTIVLVVQENRSFDHMLGWMKSLNNNINGVTGGESNPLSASNPSGGRVFFGDGSGYSEPDPGHSLEATHEQLYGAALTSTTFSSNSNDNIRIPTMEGFAEQAESINKGMSDIVMNGFKPEMVPVYKELVEEFAVCDRWFSSIPTLTQPNRLYIHSATSHGATENDTSTLIKGYPQKTIFESLEEGGCTFGIYHQYPPNTLFFRNMRKLKYLDNYHQFDLQFKDHCEKGKLPNYVVIENRYFDTKLLPGNDDHPAHDVSEGQKFVKQVYEALRSSPQWNEMLFLIIYDEHGGFYDHVPPPVTGVPSPDDIDGPEPYKFKFDRLGVRVPVIMISPWIERGTVLHGPSGPYPTSEFEHSSLAATIKKIFNLKDFLTKRDAWAGTFECVLNRSSPRQDCPEKLSEPVKMRDFEPNEDEAKLTDYQGEMVQMAATLNGDHNKDIYPHKLVENMTVSEAVKYVGDAYKVYCDECSKAKESGADEHHVVSFEALVPKSSPKSFGQKLFSCLICDN
