# Supplementary material for: Characterization of the Glehnia littoralis Non-specific Phospholipase C Gene GlNPC3 and Its Involvement in the Salt Stress Response
Source: Front Plant Sci. 2021 Dec 9;12:769599. doi: 10.3389/fpls.2021.769599 (PMC8695444; doi:10.3389/fpls.2021.769599)
Supplement: Supplementary file 4 [file Data_Sheet_2.DOCX]

**Fragments used in VIGS**

***GlNPC3***

199 bp

TGAACGGATTCAAACCAGAAATGGTGCCAGTTTACAAAGAATTGGTGGAAGAGTTTGCGG

TTTGTGACAGGTGGTTTTCCTCGATTCCGACACTAACACAGCCTAACAGATTGTACATAC

ATTCGGCTACGTCTCATGGTGCAACAGAAAATGATACAAGTACGTTGATTAAAGGGTATC

CACAGAAAACCATCTTTGA

200 bp

TTATCATCAATATCCTCCCAACACACTCTTCTTTAGGAATATGAGAAAGTTAAAGTACTT

GGACAATTACCATCAGTTTGATCTACAGTTCAAAGACCACTGTGAAAAAGGGAAATTACC

AAATTATGTGGTAATTGAAAACCGTTATTTTGACACAAAATTGTTACCCGGAAATGATGA

TCACCCTGCTCATGATGTTT

***GlPDS***

409 bp

GTCGCTAAACTTTATCAATCCAGACGAACTTTCGATGCAATGTGTATTGATTGCTTTGAACCGATTTCTTCAGGAGAAGCATGGTTCAAAGATGGCTTTCTTGGATGGAAGTCCTCCAGAAAGACTTTGCATGCCGATAGTTGATCACATACAGTCACTGGGTGGTGAAGTTCATCTCAATTCACGAGTACAGAAGATCTCTTTAAATAAAGATCATACTGTTAAGAGTCTATTACTAACCAATGGGAAGGTTATTGAAGCAGATGCATATGTAATTGCTGCTCCAGTTGATATCCTAAAGCTACTTGTGCCTGAAGAGTGGAGAGAGATTCCATACTTCAAGAAGTTGGATAAATTAGTTGGAGTTCCAGTAATCAATGTTCACATATGGTTCGACAGGAAACTGAAG
