# Supplementary material for: Characterization of the Glehnia littoralis Non-specific Phospholipase C Gene GlNPC3 and Its Involvement in the Salt Stress Response
Source: Front Plant Sci. 2021 Dec 9;12:769599. doi: 10.3389/fpls.2021.769599 (PMC8695444; doi:10.3389/fpls.2021.769599)
Supplement: Supplementary file 5 [file Data_Sheet_3.pdf]

Supplementary Figures

A

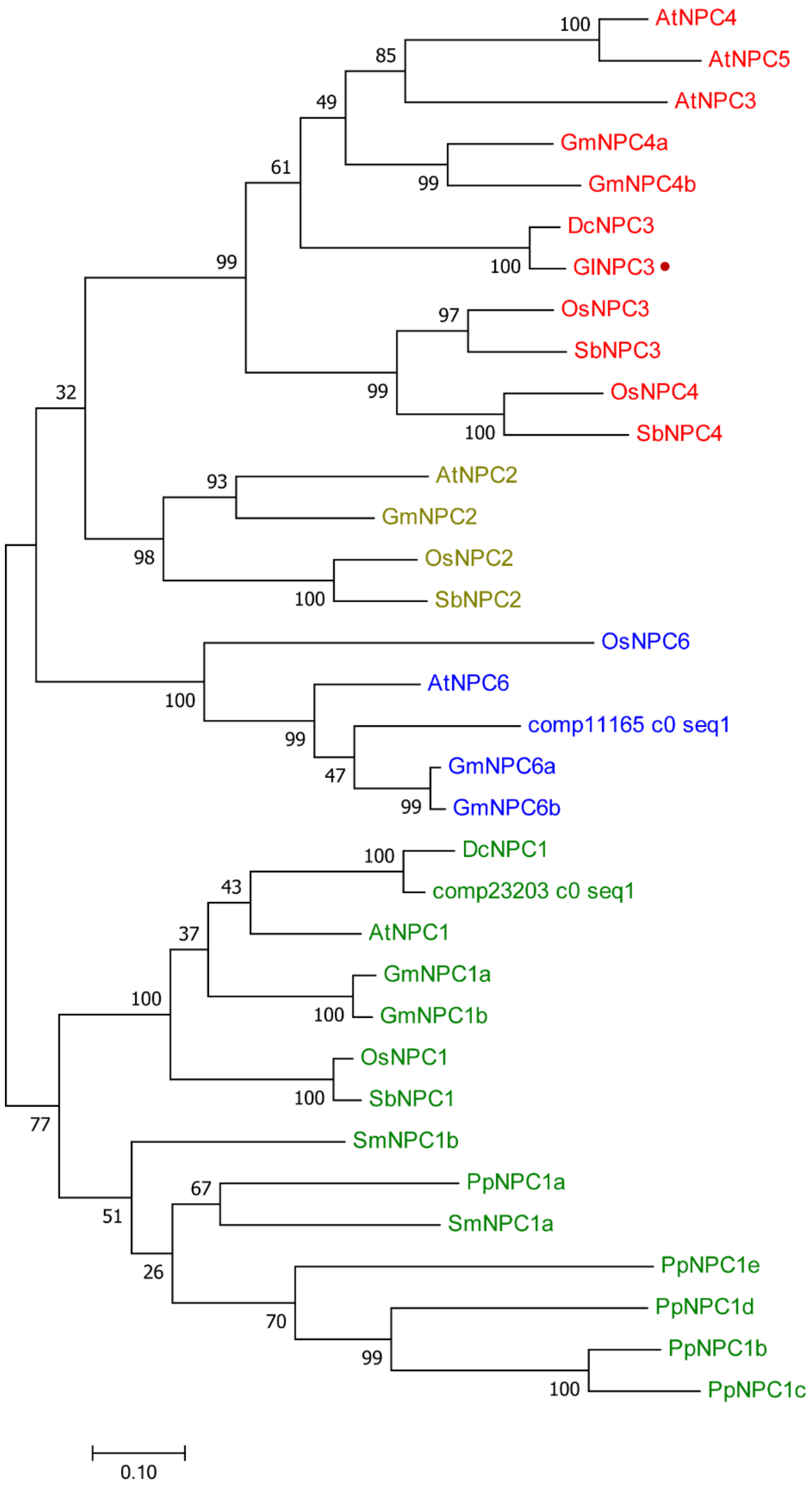

B

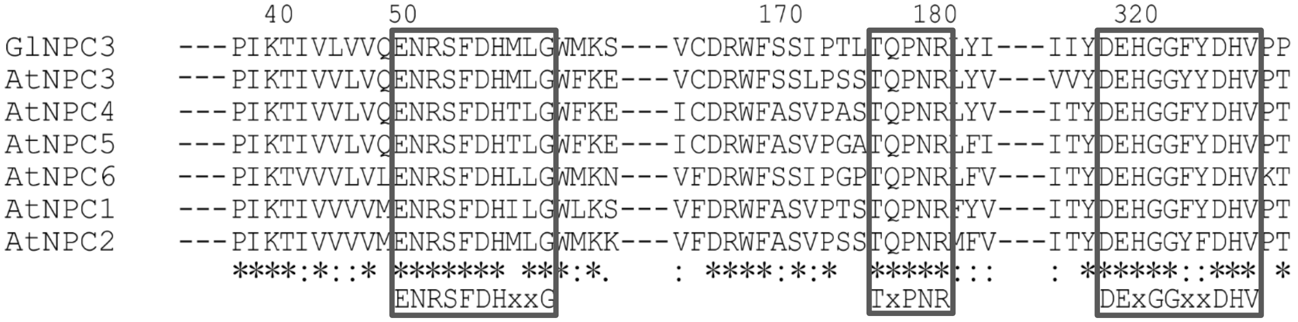

Figure S1. Bioinformatics features of *GlNPC3*.

(A) Phylogenetic analysis of plant NPCs from different species. A maximum likelihood tree was constructed using MEGA V7.0 (1,000-bootstrap, Poisson model, Partial deletion, Site coverage cutoff 50%). At, *Arabidopsis thaliana*; Gm, *Glycine max*; Os, *Oryza sativa*; Dc, *Daucus carota*; Pp, *Physcomitrium patens*; Sb, *Sorghum bicolor*; Sm, *Selaginella moellendorffii*. Based on the transcriptome data of *G. littoralis*, comp23203_c0_seq1 and comp11165_c0_seq1 are unigenes belong to *NPC* family. The transcriptome data of *G. littoralis* are deposited into National Center for Biotechnology Information (NCBI) Sequence Read Archive (SRA) (accession number: SRX547159). Accession numbers of proteins in NCBI database (https://www.ncbi.nlm.nih.gov/) are listed in Table S2.

(B) Alignment of GlNPC3 protein sequence with the *A. thaliana* NPCs. The residues in the rectangles are conserved motifs. The amino acid sequences were aligned using ClustalW2 (http://www.ebi.ac.uk/Tools/clustalw2/index.html), and the conserved domains were identified using Structure (http://www.ncbi.nlm.nih.gov/Structure/cdd.shtml).

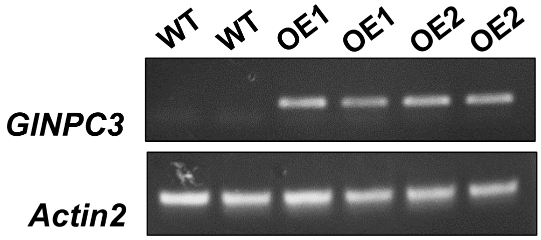

Figure S2. Level of *GlNPC3* transcript in WT and *GlNPC3*-OE plants.

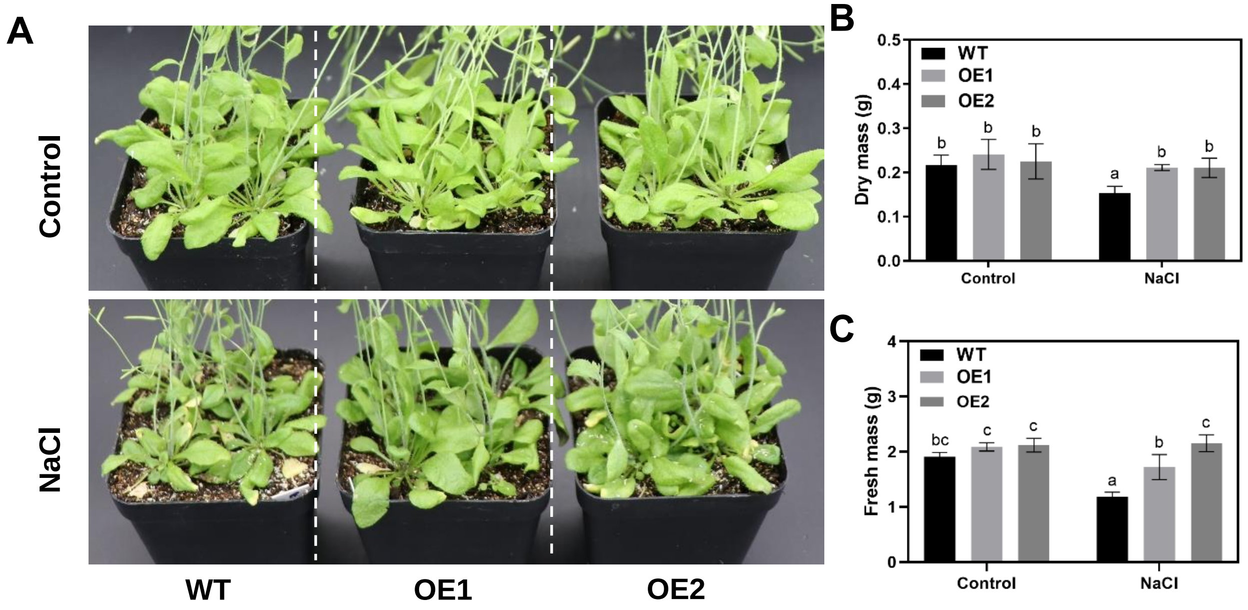

Figure S3. Phenotypes of soil-grown plants under the salt treatment.

(A) Phenotypes of the WT and *GlNPC3*-overexpressing plants during salt stress.

The 4-week plants were watered with Hoagland's culture solution containing 100 mM NaCl for 10 d. Photographs were taken at the end of the experiment.

Dry mass (B) and fresh mass (C) of the soil-grown plants under the salt treatment.

Data represent means ± SD from three biological replicates (three plants per replicate). Different letters above each bar indicate significant differences ($P < 0.05$, Duncan's multiple range test).

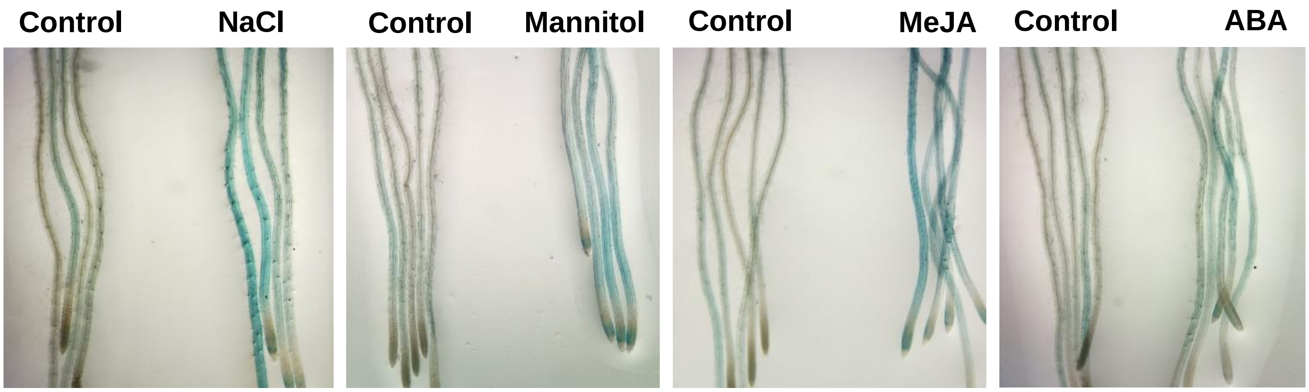

Figure S4. GUS expression in transgenic seedlings respond to NaCl, mannitol, and hormones treatments.

5-day-old seedlings grown on 1/2 MS medium were transferred to medium containing 100 mM NaCl, 300 mM mannitol, 25 μM ABA, or 100 μM MeJA respectively for 2 h. Then incubate seedlings in staining solution at 37℃ for 2 h.

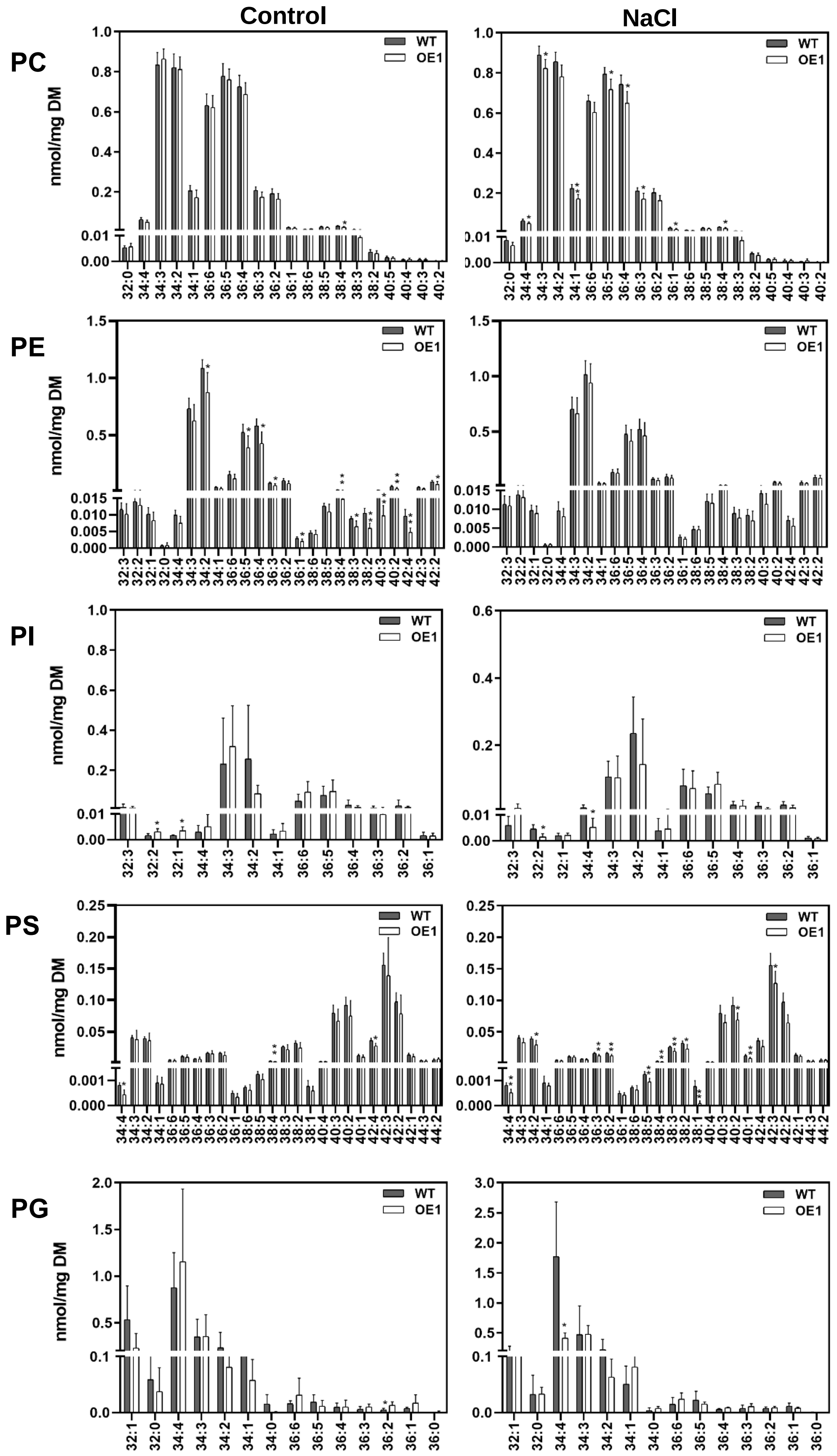

Control
NaCl
PC
PE
PI
PS
PG
nmol/mg DM
WT
OE1

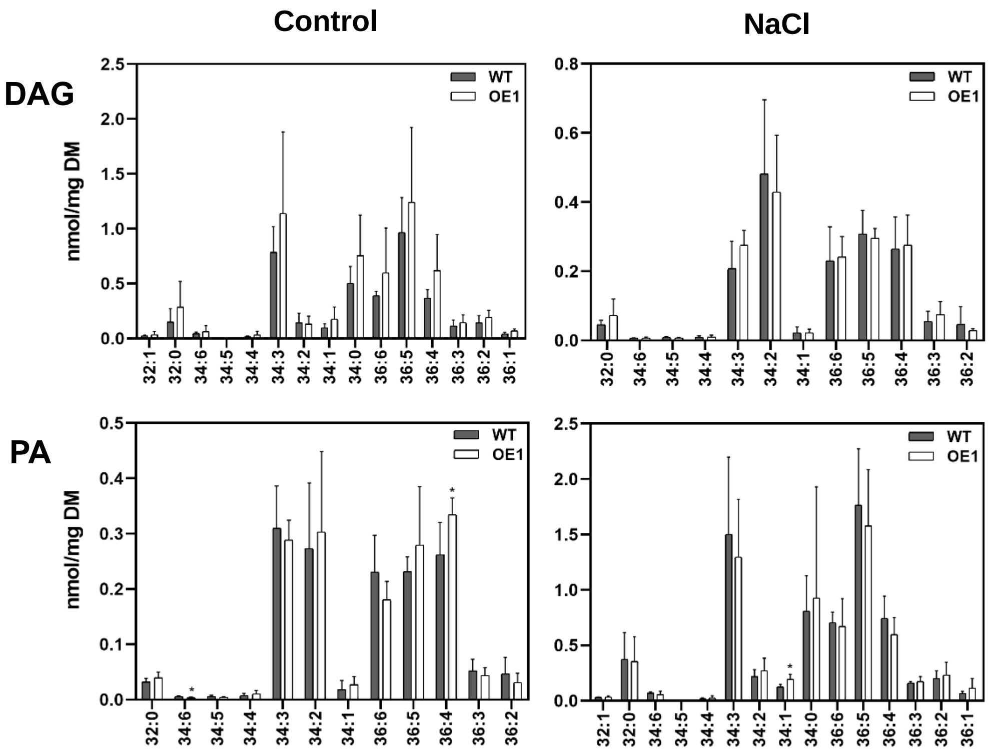

Figure S5. Comparison of changes in lipid molecular species between the WT and OE1 plants.

Molecular species are presented as a: b, where “a” represents the number of carbons, and “b” the number of double bonds. Asterisks indicate significant differences from WT: *, $P \leq 0.05$; **, $P \leq 0.01$, Duncan’s multiple range test.

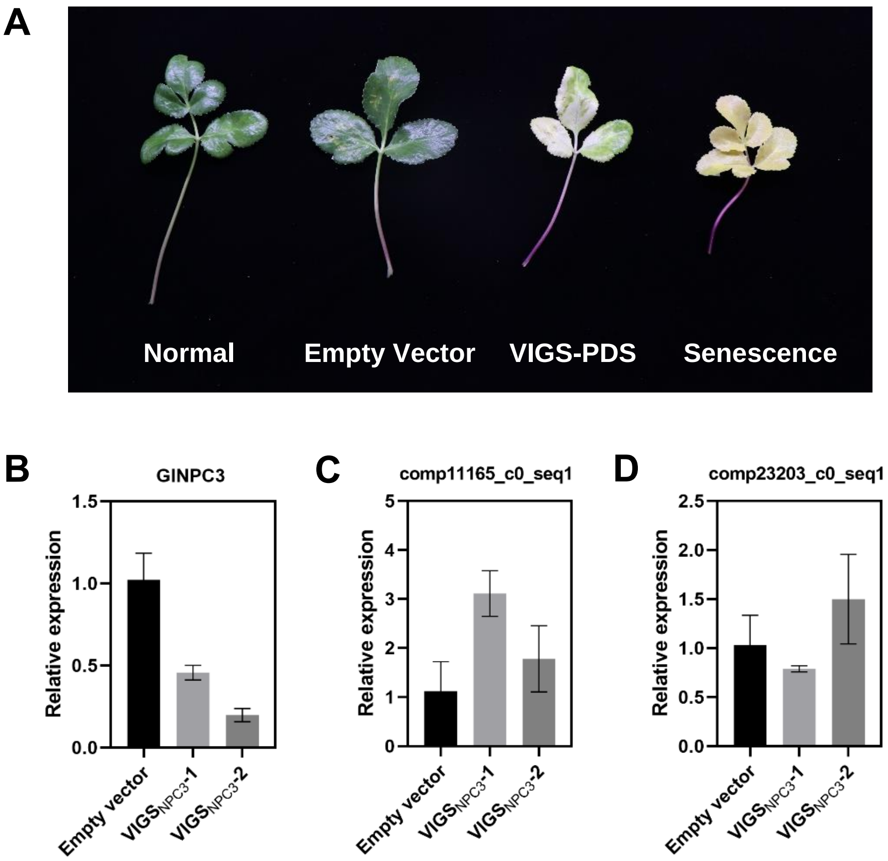

Figure S6. VIGS-mediated gene silencing in *G. littoralis* leaves.

(A) VIGS-mediated *GlPDS* gene silencing phenotype (photo-bleaching) in *G. littoralis* leaves.

(B) Comparison of *GlNPC3* expression between WT and VIGS-mediated *GlNPC3*-silenced leaves ($VIGS_{NPC3}$-1 and $VIGS_{NPC3}$-2) of *G. littoralis*.

(C, D) Comparison of two *GlNPC* genes expression for checking the silence specificity. comp23203_c0_seq1, NR_tophit_description: NPC3-like; comp11165_c0_seq1, NR_tophit_description: NPC6. The genes are included in the phylogenetic tree in Figure S1. The primers are list in Table S1.
